# Reference ranges of left ventricular diastolic multimodal ultrasound parameters in stable preterm infants in the early and late neonatal intensive care admission period

**DOI:** 10.1038/s41372-025-02278-1

**Published:** 2025-05-17

**Authors:** Koert de Waal, Enrico Petoello, Edward Crendal, Nilkant Phad

**Affiliations:** John Hunter Children’s Hospital, department of neonatology, Newcastle NSW, Australia and the University of Newcastle, Newcastle, NSW Australia

**Keywords:** Diagnostic markers, Cardiovascular diseases

## Abstract

**Background:**

Diastolic dysfunction often precedes systolic dysfunction and provides opportunity for management strategies. We aim to present reference ranges for diastolic function parameters in stable preterm infants at 2 timepoints.

**Methods:**

Ultrasound scans of clinically stable preterm infants < 30 weeks gestation with no antenatal or postnatal complications were analysed for left heart size, mitral blood flows, myocardial velocities and shortening during the early (3 to 21 days) and late (corrected gestation 34 to 37 weeks) neonatal period.

**Results:**

92 early scans and 64 late scans were included. Mitral blood flow and myocardial velocities increased with augmented atrial function leading to higher EA and e’a’ ratios and with relatively high Ee’ ratio.

**Conclusion:**

We present reference values for many left ventricular multimodal diastolic ultrasound parameters in preterm infants with uncomplicated fetal and neonatal development to guide prospective studies that explore diastolic function and diastolic heart failure in preterm infants.

## Background

There are an increasing number of neonatal clinicians trained in cardiac ultrasound to assess cardiopulmonary instability in the neonatal intensive care setting [1–3]. Typical clinical situations where cardiac ultrasound is used are hypotension, birth asphyxia, persistent pulmonary hypertension of the newborn, septic shock and assessing a patent ductus arteriosus (PDA) [4]. This diagnostic approach by bedside clinicians has led to improved clinical outcomes with reduced rates of intraventricular haemorrhage and reduced mortality by targeting treatments towards the found pathophysiology [5–7].

Clinical decision-making is based on various ultrasound measurements of cardiac function, assessment of shunts and derivates of blood flows. Often a multimodality approach is used including 2D imaging, pulse wave Doppler, colour Doppler, tissue Doppler and strain rate imaging to be able to assess the different aspects of cardiovascular function and provide a comprehensive hemodynamic consultation [8].

The initial body of research in the field of neonatal hemodynamics focussed on blood flows, shunts and systolic function [9]. With increasing insight and advances in echocardiography technologies there is now a growing interest in diastolic performance, especially left ventricular (LV) diastolic function. Diastolic dysfunction often precedes systolic dysfunction and provides an opportunity for management directives in preterm infants with a PDA [10, 11], septic shock [12], extremely small for gestational age infants [13, 14] and bronchopulmonary dysplasia (BPD) [15, 16].

The aim of this study is to present reference ranges for diastolic ultrasound parameters recommended in recent algorithms to assess LV diastolic function in stable preterm infants during the early and late neonatal intensive care unit (NICU) admission period [17–19].

## Methods

This retrospective observational study was undertaken at the John Hunter Children’s Hospital in Newcastle, Australia. Ethics approval was obtained from the Hunter New England Human Research Ethics Committee to review the data of preterm infants < 30 weeks gestation admitted in the neonatal department over a 6-year epoch between 2017 and 2023. Our hospital is a tertiary centre with around 4500 deliveries per year, which also receives unwell neonates from 6 large regional hospitals and 11 rural birthing units in New South Wales.

Cardiac ultrasound scans were obtained for clinical indications or research studies using a Vivid E90 (GE healthcare, Chicago, Illinois USA) or Philips EPIQ 5 (Philips ultrasound, Andover, Massachusetts) ultrasound system and images were stored on a local image server with analysis workstation (Philips Ultrasound workspace/Tomtec TTA2 version 51.02 with Cardiac Performance Analysis version 1.4.0.156).

### Echocardiography

**Fig. 1:**
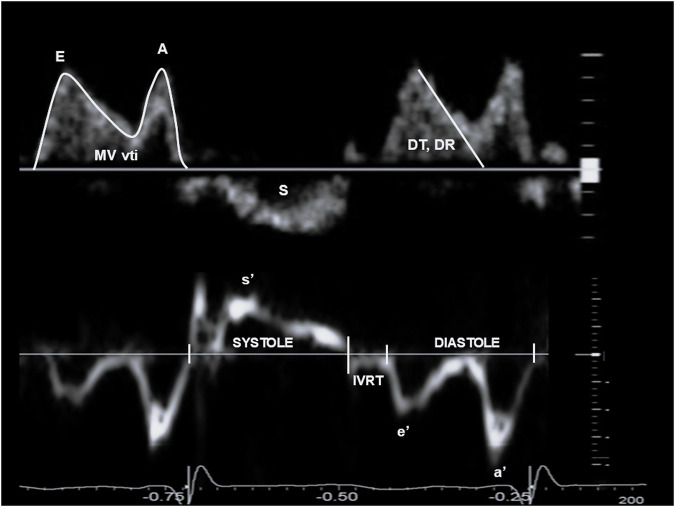
Pulse wave Doppler (top) and Tissue Doppler (bottom) trace with the various stages and physiology of the cardiac cycle and its representative ultrasound parameters. S outflow wave, MV vti mitral valve velocity time integral, E early diastolic wave, A atrial contraction wave, DT deceleration time, DR deceleration rate, s’ systolic myocardial velocity, e’ early diastolic myocardial velocity, a’ atrial contraction myocardial velocity, IVRT isovolumetric relaxation time.

**Fig. 2:**
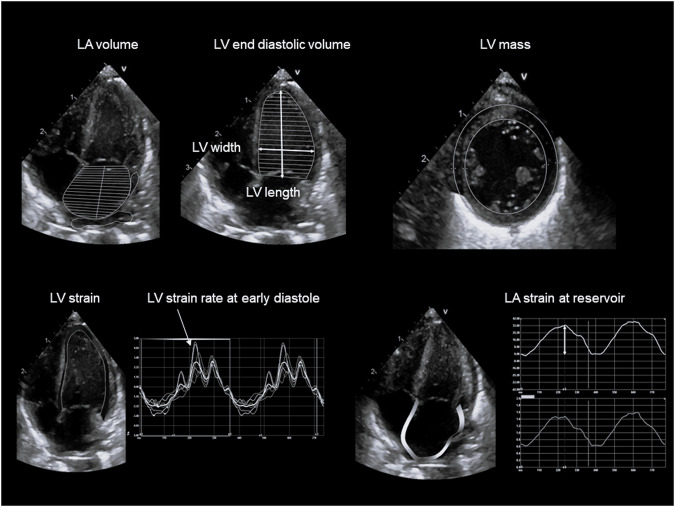
Examples of ultrasound measures of left atrial and ventricular shape and size, mass, longitudinal strain and strain rate.

Images were acquired according to the recommendations of the American Society of Echocardiography and the targeted neonatal echocardiography recommendations and saved at 100 and 200 mm/s sweep speeds [8]. Ultrasound parameters of interest for multimodal LV diastolic function assessment are presented in Table 1, Figs. 1 and 2 and included cardiac size and shape, blood flow velocities, myocardial velocities and myocardial shortening. Most measurements were derived from a 3-beat average at 200 mm/s sweep speed. Partial fusion of the diastolic wave forms is common in preterm infants. When full fusion was apparent, we ensured the infant was settled using facilitated tucking and sucrose before further image acquisition was attempted. Doppler images with persistent full fusion between the early and late diastolic wave forms were omitted from analysis. Strain measurements were taken from apical 4-chamber images in adult orientation, from one heartbeat, and analysed by one investigator using a 6-segment (LV) or 3 segment (LA) model. Images were selected based on image quality criteria such as level of foreshortening, gain settings, clarity of borders, presence of artefacts, frame rate and tracking quality. Pulmonary vein Doppler was not part of our cardiac ultrasound protocol in this epoch.

**Table 1:** Methodology of echocardiography parameters.

		Image acquisition	Image analysis
**Diastolic parameters**			
Left atrial volume (ml/kg)	LAvol	Apical 4 chamber view focussed on the left atrium	Tracing of the LA endocardial border at maximum volume, excluding pulmonary vein confluence and appendage. Monoplane method of discs, indexed to weight
Early diastolic blood flow velocity (cm/s)	E	Apical 4 chamber view with PW-Doppler at the mitral valve leaflets aligned with blood inflow	Peak velocity in early diastole
E wave deceleration time (msec)	DT	Apical 4 chamber view with PW-Doppler at the mitral valve leaflets aligned with blood inflow	Time from where peak E wave velocity is taken along the slope of deceleration extrapolated to the zero baseline
E wave deceleration rate (m/s^2^)	DR	Apical 4 chamber view with PW-Doppler at the mitral valve leaflets aligned with blood inflow	Peak E wave velocity divided by E wave deceleration time
Late diastolic blood flow velocity (cm/s)	A	Apical 4 chamber view with PW-Doppler at the mitral valve leaflets aligned with blood inflow	Peak velocity in late diastole
E to A wave ratio	EA ratio	Apical 4 chamber view with PW-Doppler at the mitral valve leaflets aligned with blood inflow	Peak E wave velocity divided by peak A wave velocity
Diastolic filling velocity time integral (cm)	MV vti	Apical 4 chamber view with PW-Doppler at the mitral valve leaflets aligned with blood inflow	Tracing of the filling wave forms in early and late diastole
Early diastolic myocardial velocity (cm/s)	e’	Apical 4 chamber view, PW-TDI on the septal annulus	Peak velocity in early diastole
Late diastolic myocardial velocity (cm/s)	a’	Apical 4 chamber view, PW-TDI on the septal annulus	Peak velocity in late diastole
e’a’ ratio	ea ratio	Apical 4 chamber view, PW-TDI on the septal annulus	Peak e’ wave velocity divided by peak a’ wave velocity
E to e’ wave ratio	Ee ratio	Apical 4 chamber view, PW-Doppler and PW-TDI. Acquire PW and TDI images close together to avoid heart rate variation	Peak E wave velocity divide by peak e’ wave velocity
Pulmonary hypertension	PH	Apical 4 chamber view or parasternal long axis view focussed on the tricuspid valve, colour doppler, short axis view at level of the papillary muscles, ductal view	Significant tricuspid regurgitation, moderate to severe septal flattening in systole, or any bidirectional or right-to-left shunt over the ductus arteriosus
Left atrial longitudinal strain at reservoir (%)	LAS_R_	Apical 4 chamber view focussed on the left atrium	Tomtec Cardiac Performance analysis v1.4. Three point and click approach at minimum volume and adjusted at maximum volume. Peak strain analysis timed to QRS
LV strain rate in early diastole (s^-1^)	SR_E_	Apical 4 chamber view focussed on the left ventricle	Tomtec Cardiac Performance analysis v1.4. Three point and click approach at minimum volume and adjusted at maximum volume. Peak strain analysis timed to QRS
E wave velocity to early diastole strain rate ratio	E/SR_E_ ratio	Apical 4 chamber view focussed on the left ventricle	E wave maximum velocity divided by peak SR_E_
Systolic time (msec)	St	Apical 4 chamber view, PW-TDI on the septal annulus	Time from then end of the late diastolic wave until the end of the positive wave after ejection
Diastolic time (msec)	Dt	Apical 4 chamber view, PW-TDI on the septal annulus	Time from the end of the positive wave after ejection until the end of the late diastolic wave
Systolic to diastolic time ratio	St/Dt ratio	Apical 4 chamber view, PW-TDI on the septal annulus	Systolic time divided by diastolic time
Isovolumetric relaxation time (msec)	IVRT	Apical 4 chamber view, PW-TDI on the septal annulus	Time from the end of the positive wave after ejection to the start of the e’ wave
**Cardiac shape and size**			
End diastolic volume (ml/kg)	EDV	Apical 4 chamber view focussed on the left ventricle	Tracing of the LV endocardial border at maximum volume. Monoplane method of discs, indexed to weight
End systolic volume (ml/kg)	ESV	Apical 4 chamber view focussed on the left ventricle	Tracing of the LV endocardial border at minimum volume. Monoplane method of discs, indexed to weight
Left ventricular length (mm)	LVL	Apical 4 chamber view focussed on the left ventricle	Distance between midpoint of the MV annulus at maximum volume and the apical endocardial border
Left ventricular width (mm)	LVW	Apical 4 chamber view focussed on the left ventricle	Distance between the septal and lateral LV wall at maximum volume
Left ventricular sphericity index	LV SI	Apical 4 chamber view focussed on the left ventricle	LV length divided by LV width
LV mass, indexed (gram/m^2^)	LV massi	Short axis view at level of the papillary muscles and the apical 4 chamber view	Area-length method using inner and outer area from short axis images and LV length from the 4-chamber view, indexed to body surface area.
LV mass to volume ratio	LV M/V	Short axis view at level of the papillary muscles and the apical 4 chamber view	LV mass divided by LV end diastolic volume
**Systolic parameters**			
LV stroke volume (ml/kg)	LV SV	Apical 4 chamber view focussed on the left ventricle	Tracing of the endocardial border at end diastole and at end systole. Monoplane method of discs, indexed to weight
LV ejection fraction (%)	EF	Apical 4 chamber view focussed on the left ventricle	Tracing of the endocardial border at end diastole and at end systole. Monoplane method of discs
Peak systolic myocardial velocity (cm/s)	s’	Apical 4 chamber view, PW-TDI on the septal annulus	Peak velocity in systole
LV peak longitudinal strain (%)	S_L_	Apical 4 chamber view focussed on the left ventricle	Tomtec Cardiac Performance analysis v1.4. Three point and click approach at minimum volume and adjusted at maximum volume. Peak strain analysis timed to QRS

### Reference patient population selection

All preterm infants with a gestational age < 30 weeks and who received at least one cardiac scan in the early NICU period were eligible. The early NICU period was defined as a postnatal age >72 h up till 21 days of age. The late NICU period was defined as a corrected gestational age (cGA) between 34 and 37 weeks. Infants with significant congenital abnormalities were excluded. Clinical data included non-invasive blood pressure measurements at the time of the scan and demographics like postnatal age and weight.

Stable preterm infants in the early NICU period were defined as having no antenatal complications that could affect fetal cardiac development such as gestational diabetes, fetal growth restriction, or prolonged (> 7 days) preterm rupture of membranes. They were born with a birth weight between the 10th and 90th percentile and with normal APGAR scores. At the time of the scan, they did not receive mechanical ventilation and the FiO_2_ was less than 25%. They had normal blood pressure and did not receive any cardiovascular medications (inotropes, steroids, inhaled nitric oxide, indomethacin, ibuprofen, paracetamol or diuretics). They also had no bradycardia or tachycardia (heart rate between 140 and 180 bpm) and on ultrasound they showed normal ejection fraction (>46%) and a closed or very small PDA (diameter < 0.5 mm). The same infants were eligible for analysis in the late NICU period if all above criteria were still met.

### Statistical analysis

Parameters are expressed as mean, standard deviation and percentiles. Simple group analyses (early versus late) were conducted using an independent *t*-test and ANOVA. Analyses were performed on GraphPad version 6 (Prism, LaJolla, CA, USA) and SPSS version 21 (IBM, Armonk, NY, USA), and *p* values < 0.05 were considered statistically significant.

## Results

During the 6-year period our unit admitted 657 preterm infants < 30 weeks gestation. Of those, 278 infants received at least one cardiac ultrasound scan in the early NICU period and a total of 389 scans were available for analysis. The flow diagram for inclusion is presented in the supplemental figure. Common reasons for exclusion were fetal growth restriction, higher oxygen requirement often in combination with the presence of a PDA, and concomitant medication use. There were 92 scans available for inclusion in the early NICU period and 64 in the late NICU period. The mean (SD) gestation was 27.6 (1.2) weeks and 51% were less than 28 weeks gestation, birth weight of 1025 (199) grams and a postnatal age of 7 [2] days at the early scan. Weight at time of the early cardiac ultrasound was 1004 (206) gram. The mean corrected gestational age at the late scan was 35.3 (0.7) weeks at a postnatal age of 59 [11] days, and a weight of 2316 (302) gram.

Feasibility was good for most diastolic parameters to average for some: PW velocities 92%, PW timings 77%, TDI velocities 94%, TDI timings 97%, volumes 99%, LV strain 81%, LA strain 85%.

**Fig. 3:**
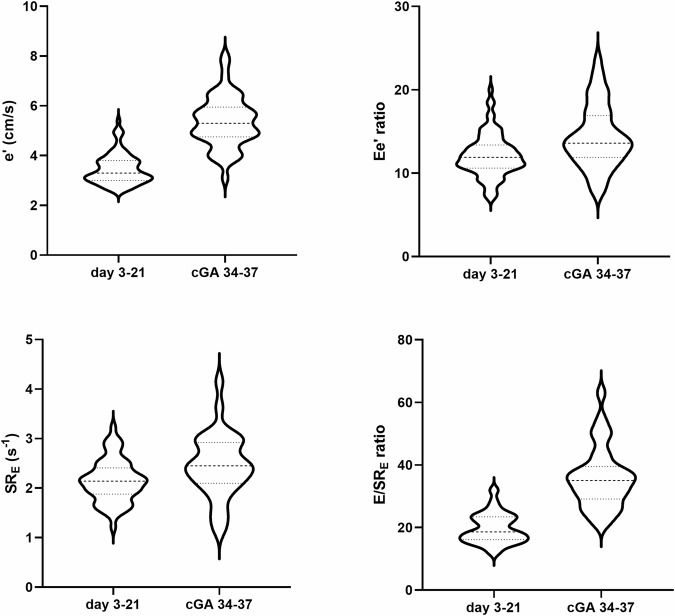
Tissue Doppler early diastolic velocities (e’), early diastolic strain rate (SR_E_) and derivates of increased left atrial pressure (Ee ratio and E/SR_E_ ratio) as violin plot. cGA, corrected gestational age in weeks.

The primary outcome with percentiles of reference ranges is presented in Table 2. Most parameters changed with increasing postnatal age. An increase in blood pressure was seen and increased mitral blood flow and myocardial velocities with augmented atrial function leading to higher EA and e’a’ ratios. In the late NICU period, a relatively high Ee ratio and E/SR_E_ ratio was found, 15 [4] and 35 [2] respectively (Fig. 3). Heart rate showed similar ranges between the early and late NICU periods but with an increased time spent in systole.

**Table 2:** Clinical and ultrasound data of stable preterm infants in the early and late NICU admission period.1.

		Early NICU period (day 3 to 21)					Late NICU period (34 to 37 weeks cGA)					*p*-value
	*percentile*	*10*	*25*	*50*	*75*	*90*	*10*	*25*	*50*	*75*	*90*	
HR	bpm	146	152	162	172	179	147	153	164	171	179	0.477
SBP	mmHg	52	55	61	66	71	63	72	76	81	86	<0.001
DBP	mmHg	29	32	36	41	44	33	35	40	44	50	<0.001
*Diastolic function*												
LAvol	ml/kg	0.58	0.71	0.86	1.03	1.24	0.72	0.83	1.00	1.14	1.33	0.001
E	cm/s	33	36	41	48	55	57	70	80	92	97	<0.001
DT	msec	57	72	83	95	108	57	82	92	113	130	0.021
DR	m/s^2^	3.9	4.4	5.6	6.3	7.3	6.7	7.3	8.5	9.4	11.1	<0.001
A	cm/s	43	51	56	62	70	76	83	91	104	116	<0.001
EA ratio		0.61	0.67	0.73	0.82	0.90	0.67	0.84	0.91	0.96	1.05	<0.001
MV vti	cm	4.4	5.2	5.7	6.6	7.9	7.5	8.7	10.0	11.0	12.1	<0.001
e'	cm/s	2.8	3.1	3.4	3.8	4.6	4.0	4.8	5.6	6.3	6.7	<0.001
a'	cm/s	4.8	5.5	5.9	6.8	7.5	6.6	7.4	8.4	9.7	10.9	<0.001
ea ratio		0.44	0.51	0.58	0.65	0.71	0.50	0.59	0.64	0.73	0.86	<0.001
Ee ratio		9.2	10.6	11.9	13.6	15.4	10.5	12.5	14.0	17.8	21.0	<0.001
LAS_R_	%	26	30	34	39	43	26	31	36	40	44	0.054
SR_E_	s^-1^	1.57	1.84	2.17	2.44	2.79	1.48	2.04	2.40	2.95	3.35	0.036
E/SR_E_ ratio		14.1	16.1	18.8	23.5	26.4	22.8	26.4	34.5	40.5	52.7	<0.001
*Cardiac cycle events*												
St	msec	164	178	195	204	215	179	201	208	215	223	<0.001
Dt	msec	148	160	177	193	209	146	157	175	193	199	0.359
St/Dt ratio		0.91	0.99	1.11	1.20	1.30	1.06	1.12	1.18	1.26	1.33	<0.001
IVRT	msec	35	40	45	55	58	38	40	44	51	55	0.777
*Cardiac shape and size*												
EDV	ml/kg	1.42	1.66	1.83	2.07	2.34	1.48	1.62	1.94	2.17	2.36	0.263
ESV	ml/kg	0.50	0.56	0.71	0.86	0.99	0.61	0.70	0.82	0.97	1.14	0.002
LVL	mm	19.3	20.9	22.1	23.4	24.5	25.4	26.7	28.7	29.3	30.6	<0.001
LVW	mm	8.7	10.2	11.4	12.7	13.6	14.5	15.3	16.6	17.4	18.1	<0.001
LV SI		1.68	1.75	1.89	2.06	2.22	1.54	1.61	1.69	1.83	1.95	<0.001
LV mass	gr/m^2^	22.9	25.8	28.7	32.3	35.1	29.0	31.2	36.1	39.5	43.0	<0.001
LV M/V		1.22	1.44	1.62	1.82	2.03	1.00	1.28	1.43	1.63	1.82	0.001
*Systolic function*												
EF	%	49	54	62	68	72	46	49	55	60	67	0.003
LV SV	ml/kg	0.79	0.90	1.13	1.31	1.53	0.72	0.87	1.03	1.23	1.44	0.393
s'	cm/s	3.3	3.7	4.0	4.3	4.7	4.4	4.8	5.5	6.0	6.5	<0.001
S_L_	%	15.3	16.1	17.2	19.0	21.1	17.4	18.5	19.4	21.2	22.3	<0.001

Data presented as percentiles. cGA, corrected gestational age. Abbreviations are provided in Table .

## Discussion

This study presents reference ranges of multimodal ultrasound parameters to assess LV diastolic function in a large cohort of stable preterm infants at 2 specific time where clinical assessments are frequently made during a NICU admission, either for acute deterioration (eg PDA, sepsis) or for screening before transfer or discharge. We found changes with increasing postnatal age with increased mitral inflow and atrial predominance like what was found in earlier cohorts. Di Maria et al. studied a large cohort of preterm infants <34 weeks around day 7 and at 36 weeks corrected gestational age. All admitted infants were eligible and thus included infants with a PDA, mechanical ventilation and those who later developed BPD [20]. They found that maturational changes in TDI velocities were independent of GA at birth and also of common neonatal complications. However, the study did feature a substantial number of significant outliers that could represent individual changes in diastolic function, and only reported pulse wave and tissue Doppler findings. LV diastolic function has a variety of determinants that are best captured with a multimodal approach with added strain measurements to detect changes in relaxation, recoil, stiffness and LA function simultaneously [21, 22].

Hirose et al. studied 30 stable preterm infants around day 28 and added strain rate analysis, with findings comparable to our data [23]. The authors compared preterm LV diastolic function to that of healthy term infants and hypothesised that preterm infants in the late NICU period have less efficient relaxation and possible increased LV stiffness when compared to healthy term infants, which could lead to impaired early filling with raised left atrial pressure. Our data showed similar findings with an increased Ee ratio and lower SR_E_ at the late scan, and we hypothesise that part of these changes is related to altered cardiac development after preterm birth. Other studies have shown that preterm infants often reveal cardiac remodelling with a dilated, hypertrophied, and a more spherical heart at 36 weeks cGA, contributing to altered diastolic function [24, 25].

Heart rate was similar in both NICU periods, which might have contributed to the relative higher left atrial pressure found in the late NICU period. Fetal heart rate tends to reduce with increasing gestational age, but this does not occur when born preterm [26, 27]. Heart rate has an important effect on diastolic function and its ultrasound parameters. Physiological heart rate changes can affect mitral inflow patterns [28], and the diastolic duration decreases as heart rate increases [29, 30]. We found that the systolic to diastolic ratio increased over time, but further studies are needed to explore how cardiac cycle events changes with significant tachycardia eg. during disease, as we excluded infants with low and high heart rate for this study.

The limitation of our study is the lack of data in very young gestational age infants. Due to their very small cardiac size and relative high heart rate, we expect that changes in diastolic parameters will be disproportional to what is presented in this cohort. We also excluded measurements during the immediate transitional period due to the expected rapid and ongoing hemodynamic changes. One would require measurements at strict set time points (eg. every 6 h) after birth and adjust for various interventions and heart lung interactions to establish reference values during transition. Our ultrasound protocol did not include pulmonary vein Doppler or propagation velocity. Current adult algorithms to assess LV diastolic function recommend adding pulmonary vein Doppler if there are insufficient criteria to diagnose diastolic dysfunction or in special populations [17, 31]. In children with congenital heart disease, a machine learning model analysed ultrasound data and invasive measures of relaxation, end diastolic pressure and contractility [32]. Propagation velocity correlated most with relaxation, and strain measures performed well for LV filling pressure. Diastolic function is complex, and no single ultrasound parameter is likely to be able to accurately assign risk profiles [18, 19]. A multimodal approach, where an increasing number of parameters that fall outside the p10 or p90 range are used to identify diastolic dysfunction, will have the highest likelihood of detecting preterm infants at risk of respiratory or cardiovascular compromise during their NICU stay.

An important question that remains is which parameters to propose in an algorithm to diagnose diastolic dysfunction in preterm infants? In our previous work we proposed and tested an algorithm against signs and symptoms of heart failure in the early NICU period using LAvol, e’, Ee’ ratio, LAS_R_ and the presence of pulmonary hypertension [33]. Adult recommendations rely heavily on E and e’ as core criteria for diagnosing diastolic dysfunction [17]. In healthy adults, the mitral E remains relatively unchanged until older age [34]. On the contrary, in our study the mitral E wave nearly doubled over a 7 week period. Only IVRT and left atrial strain remained stable during the NICU period, but most other parameters changed during the NICU admission. These developmental changes will have to be incorporated in any proposed algorithm for preterm infants, likely with different criteria for the early and late NICU period.

## Conclusion

We present reference values for a large number of multimodal LV diastolic ultrasound parameters in a cohort of preterm infants with strict classifications to be considered of normal fetal and postnatal development and clinically stable. We found that cardiac postnatal growth after preterm birth with a stable NICU period led to signs of increased LV stiffness and higher LV filling pressure. Cardiac function was maintained through reliance on good LA function. The data in this study can be used to guide further prospective studies that explore diastolic heart failure in preterm infants. Diastolic heart failure is a clinical syndrome characterised by respiratory deterioration and oedema, features recognisable to the neonatologists but not often diagnosed. Further insight into neonatal heart failure could provide an opportunity for early intervention in preterm infants at risk of cardiovascular complications.

## Supplementary information

Inclusion flow diagram
