# Supplementary figures and images for: Reference ranges of left ventricular diastolic multimodal ultrasound parameters in stable preterm infants in the early and late neonatal intensive care admission period
Source: J Perinatol. 2025 May 17;45(7):920–6. doi: 10.1038/s41372-025-02278-1 (PMC12316592; doi:10.1038/s41372-025-02278-1)

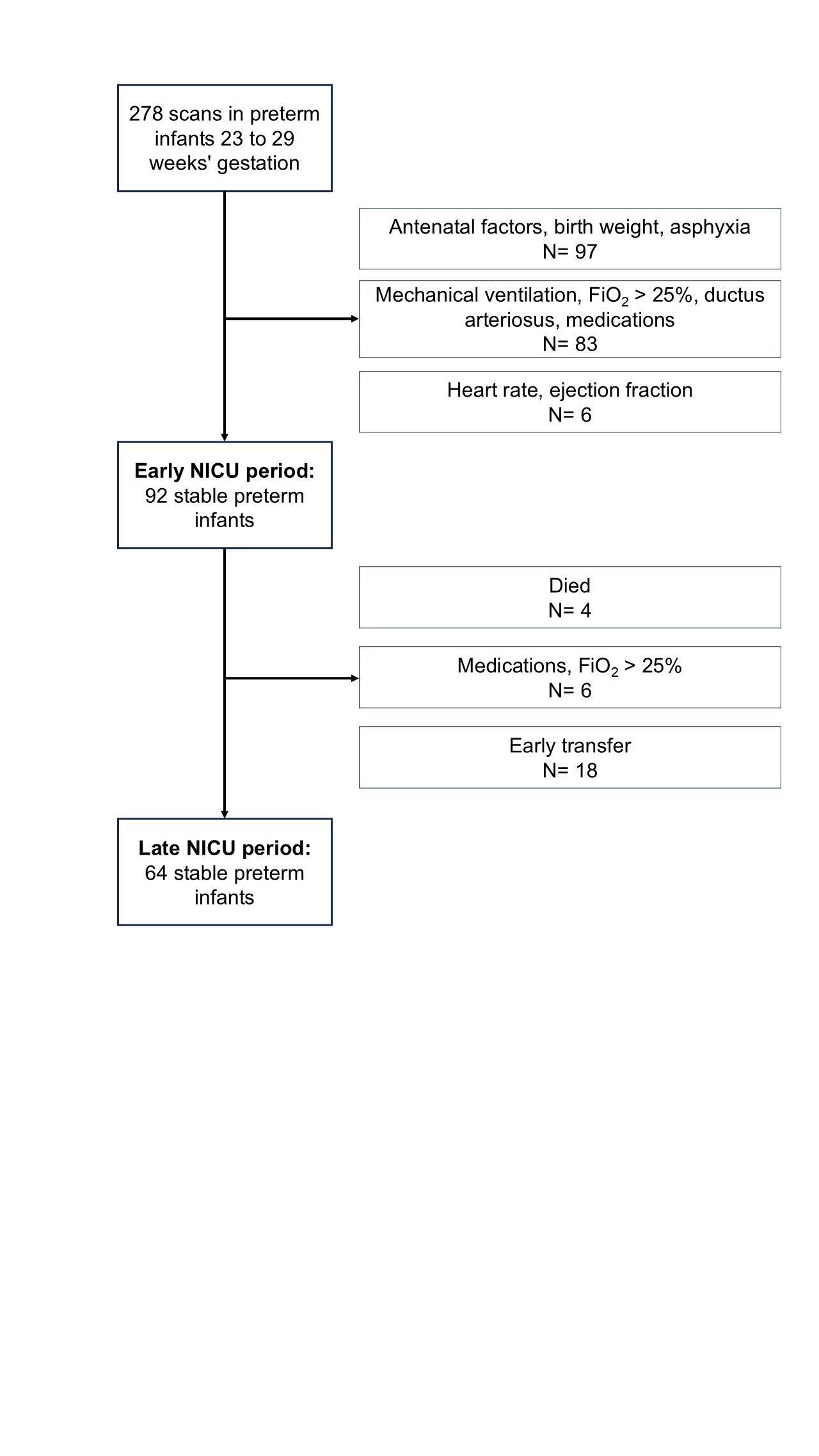

Supplement: Supplementary file 1 — Inclusion flow diagram [file 41372_2025_2278_MOESM1_ESM.bmp]
